# Tracking and blocking interdependencies of cellular BRAF-MEK oncokinase activities

**DOI:** 10.1093/pnasnexus/pgad185

**Published:** 2023-06-05

**Authors:** Jakob Fleischmann, Selina Schwaighofer, Louis De Falco, Florian Enzler, Andreas Feichtner, Valentina Kugler, Philipp Tschaikner, Roland G Huber, Eduard Stefan

**Affiliations:** Institute of Biochemistry and Center for Molecular Biosciences, University of Innsbruck, Innrain 80/82, Innsbruck 6020, Austria; Institute of Biochemistry and Center for Molecular Biosciences, University of Innsbruck, Innrain 80/82, Innsbruck 6020, Austria; Tyrolean Cancer Research Institute (TKFI), Innrain 66, Innsbruck 6020, Austria; Bioinformatics Institute (BII), Agency for Science Technology and Research (A*STAR), 30 Biopolis Street, Matrix #07-01, Singapore 138671, Singapore; Institute of Biochemistry and Center for Molecular Biosciences, University of Innsbruck, Innrain 80/82, Innsbruck 6020, Austria; Institute of Biochemistry and Center for Molecular Biosciences, University of Innsbruck, Innrain 80/82, Innsbruck 6020, Austria; Institute of Biochemistry and Center for Molecular Biosciences, University of Innsbruck, Innrain 80/82, Innsbruck 6020, Austria; Tyrolean Cancer Research Institute (TKFI), Innrain 66, Innsbruck 6020, Austria; Institute of Molecular Biology, University of Innsbruck, Technikerstrasse 25, Innsbruck 6020, Austria; Bioinformatics Institute (BII), Agency for Science Technology and Research (A*STAR), 30 Biopolis Street, Matrix #07-01, Singapore 138671, Singapore; Institute of Biochemistry and Center for Molecular Biosciences, University of Innsbruck, Innrain 80/82, Innsbruck 6020, Austria; Tyrolean Cancer Research Institute (TKFI), Innrain 66, Innsbruck 6020, Austria; Institute of Molecular Biology, University of Innsbruck, Technikerstrasse 25, Innsbruck 6020, Austria

**Keywords:** MAPK, cancer mutation, drug efficacy, combination therapy, precision medicine

## Abstract

The selective targeting of mutated kinases in cancer therapies has the potential to improve therapeutic success and thereby the survival of patients. In the case of melanoma, the constitutively active MAPK pathway is targeted by a combinatorial inhibition of BRAF and MEK activities. These MAPK pathway players may display patient-specific differences in the onco-kinase mutation spectrum, which needs to be considered for the design of more efficient personalized therapies. Here, we extend a bioluminescence-based kinase conformation biosensor (KinCon) to allow for live-cell tracking of interconnected kinase activity states. First, we show that common MEK1 patient mutations promote a structural rearrangement of the kinase to an opened and active conformation. This effect was reversible by the binding of MEK inhibitors to mutated MEK1, as shown in biosensor assays and molecular dynamics simulations. Second, we implement a novel application of the KinCon technology for tracking the simultaneous, vertical targeting of the two functionally linked kinases BRAF and MEK1. Thus, we demonstrate that, in the presence of constitutively active BRAF-V600E, specific inhibitors of both kinases are efficient in driving MEK1 into a closed, inactive conformation state. We compare current melanoma treatments and show that combinations of BRAFi and MEKi display a more pronounced structural change of the drug sensor than the respective single agents, thereby identifying synergistic effects among these drug combinations. In summary, we depict the extension of the KinCon biosensor technology to systematically validate, anticipate, and personalize tailored drug arrangements using a multiplexed setup.

## Introduction

**Fig. 1.**
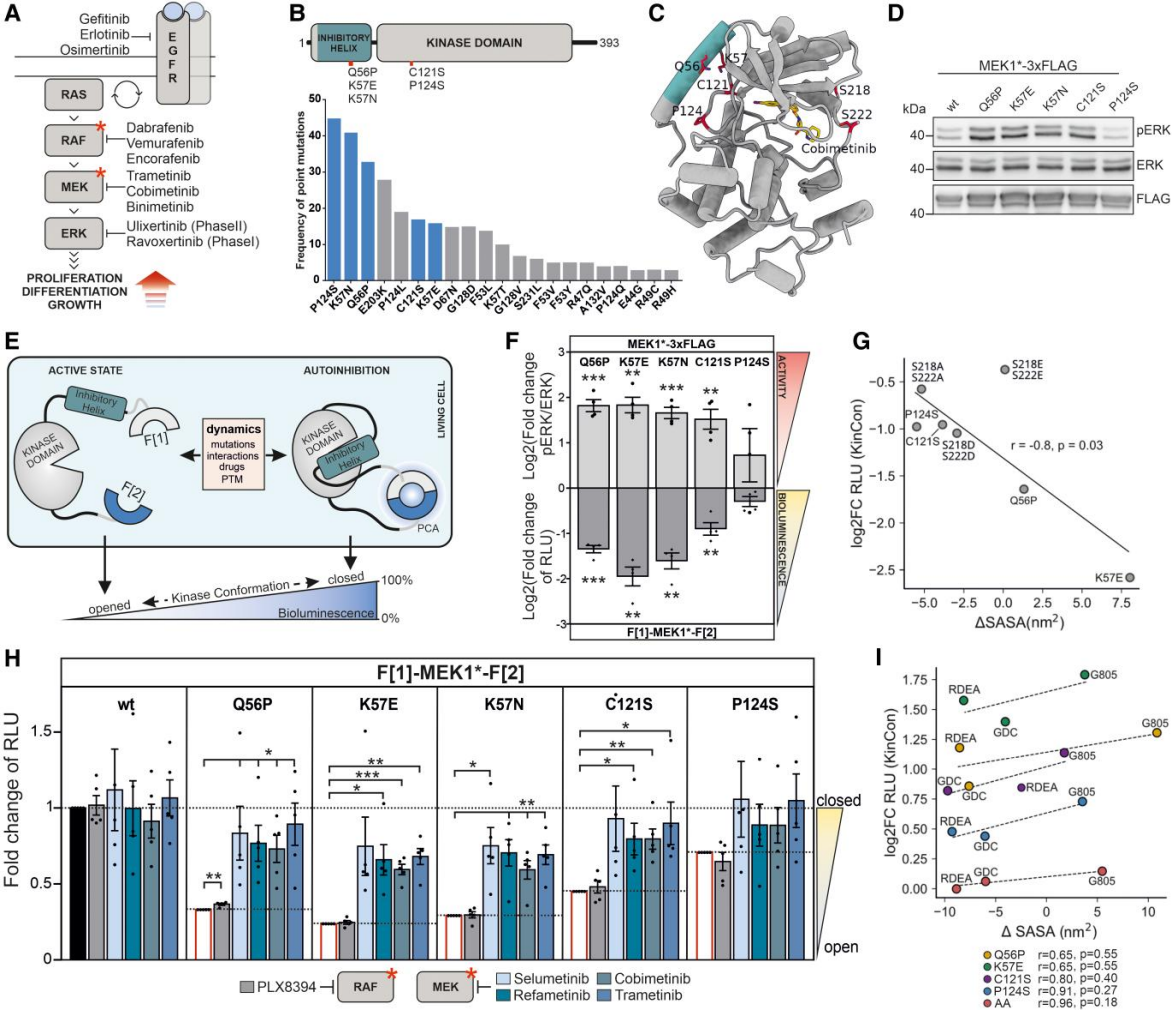
Mutation and inhibitor-induced effects on MEK1 conformation in cells and in silico. A) Overview of the MAPK pathway and indication of selected kinase inhibitors. B) MEK1 domain organization showing the N-terminal inhibitory helix A and kinase domain. MEK1 patient mutations are highlighted. The respective mutation frequencies are indicated (COSMIC database) and selected ones investigated. C) 3D model of human MEK1 bound to Cobimetinib (derived from PDB 1SJ9 and 3EQI). The inhibitory helix A is colored. MEK1 sites S218/S222 (RAF phosphorylation) are indicated. D) ERK1/2 phosphorylation upon overexpression of the indicated MEK1-triple-Flag fusion proteins. One representative western blot out of *n* = 4 independent experiments is shown. Statistical evaluation is included in F). E) Schematic representation of the MEK1 kinase conformation (KinCon) reporter, fragments of the *Renilla* luciferase are indicated with F[1] and F[2]. Inhibitor binding, protein–protein interactions (PPI), patient mutations, or posttranslational modifications (PTM) convert the MEK1-KinCon reporter into different kinase conformation states, altering the PCA-emitted bioluminescence signals. F) Effect of patient mutations on MEK1-KinCon dynamics and pERK level. Bars represent log2 of the mean of mutation-induced elevation of the pERK/ERK ratio and reduction in the bioluminescence signal, respectively. Bioluminescence was measured 48 h post transfection, normalized to expression levels determined by western blot and compared with wt MEK1 (±SEM, *n* = 4). HEK293T lysates expressing the corresponding MEK1 construct were subjected to western blotting and the pERK/ERK ratio was determined relative to the wt MEK1 pERK/ERK ratio (±SEM, *n* = 4). Further, a significant negative correlation between RLU and pERK levels was determined with a two-tailed nonparametric Spearman correlation test (*P < 0.0167). G) Log2 of the data of *n* = 3 shown in F) is blotted against the change in solvent accessible surface area (SASA). The change in maximum SASA of MEK1 mutants as measured by MD simulation is negatively correlated with the change in RLU as measured by the KinCon reporter assay. H) Determination of structural rearrangements of mutant MEK1-KinCon reporters after exposure to 1 µM of indicated MEKi, PLX8394, or DMSO for 1 h. Measured bioluminescence signals were normalized to the respective DMSO control of each KinCon reporter and adjusted to the levels of untreated samples (data from F lower part before log2 transformation) and (±SEM, *N* = 5). I) Log2 representation of the data of *n* = 4 shown in H) is blotted against the change in solvent accessible surface area (SASA). The difference in maximum SASA between wt and mutant is positively correlated with a given mutant's change in RLU. Statistical significance for F and H) One-sample *t*-test (*P < 0.05, **P < 0.01, ***P < 0.001). MEKi: RDEA, Refametinib; GDC, Cobimetinib; G805, Selumetinib; Trametinib.

Kinase inhibitor treatments have become a prime example of personalized cancer therapies by selectively targeting the respective mutated kinase entity (1, 2). Initially, kinase blockers are effective before drug resistance occurs (3, 4). Thus, more efficient BRAF and MEK inhibitor (BRAFi/MEKi) combinations emerged (Fig. 1A) (5, 6). However, the diversity of kinase mutations emphasizes the need for additional personalized drug assessments to rapidly anticipate and specify the most promising drug combination (7, 8).

## Results and discussion

MEK1 patient mutations are rare and located within, or in the structural vicinity of the N-terminal inhibitory kinase helix (Fig. 1B) (9). This autoinhibitory module acts as a negative regulatory region stabilizing the inactive MEK1 conformation (Fig. 1C) (10). We validated the listed MEK1 mutations, and their pathway activation, showing that Q56P, K57E, K57N, and C121S mutations increased ERK1/2 phosphorylation (Fig. 1D and F, upper part). Next, we determined the consequences of mutations, kinase activation, and small-molecule binding on MEK1 activity conformations using wild-type (wt) and mutant MEK1-KinCon biosensors (Fig. 1E), in quantifications of intramolecular and full-length kinase conformation rearrangements in intact cells (7, 11). The MEK1 mutations Q56P, K57E, K57N, C121S, and less pronounced P124S, converted the KinCon biosensor to a more opened and thus active conformation, represented by a lower bioluminescence signal (RLU) (Fig. 1F, lower part). The tested MEK1 mutations lead to elevated downstream signaling, which is reflected by an inverse correlation with the corresponding mutant MEK1 biosensor (Fig. 1F, upper part). The full-length structure model of human MEK1 (Fig. 1C) was used in molecular dynamics (MD) simulation experiments validating the influences of patient mutations on the MEK1 conformation, in addition to functional mutations already investigated (12). The changes in signal strength shown in Fig. 1F negatively correlate with the change in maximum solvent accessible surface area (SASA). The increase in SASA is predictive of the opened MEK1 conformation (Fig. 1G). Several MEKis act allosterically, thereby strengthening the binding of the N-terminal inhibitory helix to the activation site and locking the kinase in a catalytically inactive state (10). We exposed HEK293T cells expressing the indicated MEK1-KinCon reporters to the MEKi Selumetinib, Refametinib, Cobimetinib, and Trametinib. The BRAFi PLX8394 was used as a negative control (7, 12). The bioluminescence data were normalized to the values of the untreated samples shown in Fig. 1F (lower part) before their log2 transformation. All MEKi promoted a structural rearrangement of the mutated MEK1-KinCon reporters from a more opened, back to a more closed and inactive MEK1 kinase conformation (Fig. 1H). In agreement with KinCon reporter measurements (alterations of RLU), the binding of MEKi reverses the opening effect in the MD simulations, particularly for Cobimetinib (GDC0793) and Refametinib (RDEA119) (Fig. 1I). The biosensor data are in accordance with the MD simulations. The elevated phospho-ERK levels confirm that the tested patient mutations displace the N-terminal inhibitory helix, resulting in a more opened kinase conformation. Drug binding reverses this opening effect of the mutants, which leads to an increase in RLU and a decrease in SASA, respectively.

**Fig. 2.**
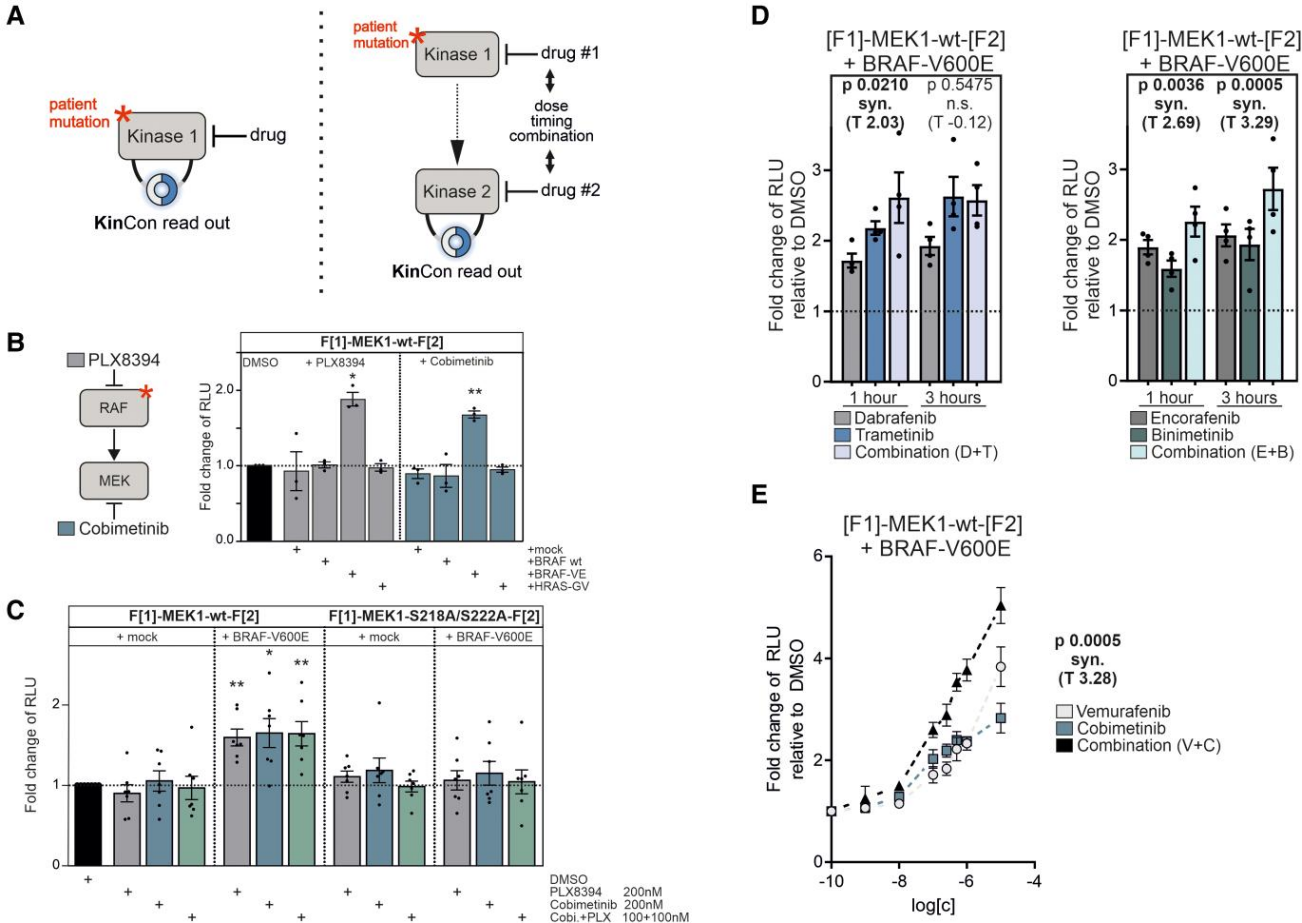
RAF-MEK interdependencies in cells tracked using the KinCon technology. A) KinCon reporter dynamics can be measured at different levels of a signal cascade. (left) The direct impact of a mutation and/or inhibitor. (right) Mutations/drugs impact on kinase 1 which affects kinase 2. The interplay of both drugs on the kinase structures is recorded. B) Effect of 1 µM treatment of Cobimetinib or PLX8394 for 1 h on MEK1-KinCon reporter dynamics in the presence of BRAF, BRAF-V600E, HRAS-G12V, or empty vector. Bars represent the mean of measured bioluminescence signals relative to DMSO (±SEM, *n* = 3). C) Dose-dependent effect of a 1 h inhibitor treatment on indicated MEK1-KinCon dynamics in the presence and absence of BRAF-V600E. Cells were treated as indicated schedules. Bars represent the mean bioluminescence signals relative to DMSO (±SEM, *n* = 7). D) Impact of MEKi and BRAFi combinations on the wt MEK1-KinCon in the presence of BRAF-V600E or mock. Single agents (100 nM) or drug combinations (100 nM each; total 200 nM) were applied for 1 or 3 h. Bars represent the mean bioluminescence signals relative to DMSO (±SEM, *n* = 4). E) Impact of dose-dependent Vemurafenib, Cobimetinib, or respective combination treatment on the wt MEK1-KinCon in the presence of BRAF-V600E. Symbols represent the mean bioluminescence signals relative to DMSO at increasing inhibitor concentrations on a logarithmic scale (±SEM, *n* = 6). The Min test was used in panels D and E to assess the synergistic effect of a drug combination relative to the effects of the combination's constituent drugs (*P* < 0.05 synergism). Unless otherwise specified, a one-sample *t*-test was used to evaluate statistical significance (*P < 0.05, **P < 0.01, ***P < 0.001).

MEKis are administered in wt MEK and BRAF-V600E mutant cancers. However, no KinCon dynamics for wt MEK1 were observed. We expanded the KinCon reporter setup to tackle inhibitor-driven conformation alterations caused by the vertical targeting of the interlinked kinases (Fig. 2A). In co-expression experiments, we showed that just in the presence of active BRAF-V600E PLX8394 and Cobimetinib, the wt MEK1-KinCon reporter was converted back into a more closed conformation (Fig. 2B). In the presence of BRAF-V600E, drug-induced effects on wt MEK1-KinCon dynamics were recorded at indicated nanomolar concentrations for PLX8394 and Cobimetinib and when applied as a combinatorial treatment (Fig. 2C). In accordance, no change in bioluminescence was observed using the phosphorylation-deficient S218A/S222A MEK1-KinCon (Fig. 2C). This underscores that the phosphorylated and opened MEK1 conformation is more accessible for small-molecule inhibition.

The three indicated combinations of BRAFi/MEKi are approved for the treatment of melanoma (13). Cells co-expressing the wt MEK1-KinCon reporter and BRAF-V600E were treated with single agents or indicated inhibitor combinations for 1 or 3 h (Fig. 2D and E). For unveiling synergistic effects of the different combinations over the respective single agents, a Min test over the obtained bioluminescence signal changes was carried out, where *P* < 0.05 indicates drug synergism (14, 15). The Encorafenib/Binimetinib combination showed synergistic effects for 1 and 3 h treatment. In contrast to synergistic effects for Dabrafenib/Trametinib for 1 h, it was not evident for Vemurafenib/Cobimetinib with just one concentration tested (Fig. 2D and Table S1). Drug exposure had no effect in the absence of BRAF-V600E (Table S1). Consequently, the drug profiling experiments were repeated with increasing drug concentrations of Vemurafenib/Cobimetinib. Using this strategy, we revealed synergistic effects of the tested combination over the single agents (Fig. 2E). Thus, the presented reporter multiplex setup allows for dose-dependent monitoring of the inhibition of either kinase or both in living cells.

Here, we have extended a biosensor concept aiming to track interconnected kinase activity states. We provide a biosensor framework for predicting and depicting the efficacy of drugs applied in the vertical targeting of the oncogenic MAPK pathway. The inhibition profiles of two drug targets were simultaneously recorded in the intact cell setting. KinCon reporters of key gatekeeper kinases or decisive signaling nodes can become the core readout in multiplexing approaches to systematically validate drug combination efficacies (Fig. 2A).

## Materials and methods

### Cell culture

HEK293T cells were used as previously described (12).

### Expression constructs

The MEK1-KinCon reporter was generated as described in Mayrhofer *et al*. (7) and Röck *et al*. (11). The Flag-tag was inserted C-terminally of MEK1 and N-terminally of BRAF-V600E, respectively.

### Luciferase protein-fragment complementation assay analyses

Forty-eight hours post transfection, cells were exposed to drug treatments. The bioluminescence was measured using the PHERAstar FSX (BMG Labtech).

### Preparation of cell lysates and ERK phosphorylation

Forty-eight hours post transfection, cells were lysed in RIPA lysis buffer. Protein levels were determined using the appropriate antibodies (listed in Supporting Information).

### Molecular dynamics preparation

Apo MEK1 models were derived from PDB structures 1SJ9 and 3EQI, while ligand-bound systems were prepared using relevant crystal structures (4U7Z, 4LMN, and 3E8N for MEK1:G805, MEK1:GDC, and MEK1:RDEA, respectively). MEK1 mutants were produced with PyMOL's mutagenesis tool (Delano, WL, 2002, https://www.scirp.org/(S(vtj3fa45qm1ean45vvffcz55))/reference/ReferencesPapers.aspx?ReferenceID=1958992).

### Simulation setup and analysis

Simulations were performed using GROMACS 2019.3. MEK1 SASA, RMSD, and radius of gyration (*R*_g_) were performed on the fitted and truncated trajectory and measured using the corresponding GROMACS modules.

Supporting Information provides extended materials and methods.

## Supplementary Material

pgad185_Supplementary_Data

## Data Availability

Data supporting the findings of this study are available within the article and the corresponding raw data will be freely available as a PDF-File on Zenodo.
