# Supplementary material for: Tracking and blocking interdependencies of cellular BRAF-MEK oncokinase activities
Source: PNAS Nexus. 2023 Jun 5;2(6):pgad185. doi: 10.1093/pnasnexus/pgad185 (PMC10267685; doi:10.1093/pnasnexus/pgad185)
Supplement: pgad185_Supplementary_Data [file pgad185_supplementary_data.docx]

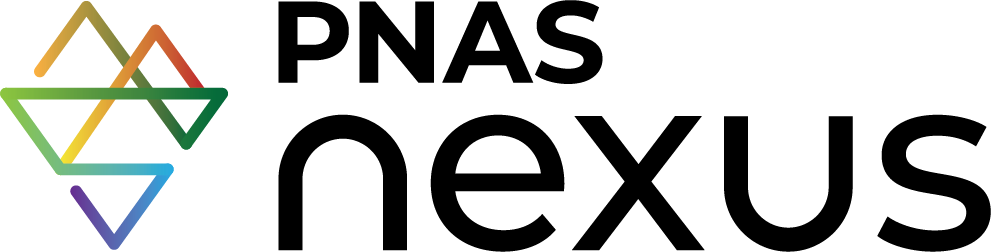


**Supporting Information for**

Tracking and blocking interdependencies of cellular BRAF-MEK oncokinase activities here.

Jakob Fleischmann^1 $^, Selina Schwaighofer^1,2, $^, Louis De Falco^3^, Florian Enzler^1^, Andreas Feichtner^1^, Valentina Kugler^1^, Philipp Tschaikner^2,4^, Roland G Huber^3^, Eduard Stefan^1,2,4*^

^1^Institute of Biochemistry and Center for Molecular Biosciences, University of Innsbruck, Innrain 80/82, 6020 Innsbruck, Austria
^2^Tyrolean Cancer Research Institute (TKFI), Innrain 66, 6020 Innsbruck, Austria
^3^Bioinformatics Institute (BII), Agency for Science Technology and Research (A*STAR), Singapore 138671, Singapore*

^4^Institute of Molecular Biology, University of Innsbruck, Technikerstrasse 25, 6020 Innsbruck, Austria.

**Email of the corresponding author:**  eduard.stefan@uibk.ac.at; Tel.: 0043 512 507 57531

**This PDF file includes:**

Extended Materials and Methods
Table S1

SI References

**Extended Materials and Methods**

**Reagents**

h-coelenterazine (Nanolight, #301); Trametinib (Synonyms: GSK1120212; JTP-74057, MedChemExpress, HY10999), Cobimetinib (Synonyms: GDC-0973; XL518, MedChemExpress, HY13064), Refametinib (Synonyms: BAY 869766; RDEA119, MedChemExpress, HY-14691), Selumetinib (Synonyms: AZD6244; ARRY-142886, MedChemExpress, HY-50706), PLX8394 (MedChemExpress, HY18972).

**Cell culture and antibodies**

HEK293T cells were obtained from the clinical department of Freiburg University (Thien et al, 2015). HEK293T cells were validated by DSMZ for their origin using short tandem repeat analysis. Cells are tested regularly for mycoplasma by PCR using suitable primers and/or Universal Mycoplasma Detection Kit (ATCC #30-1012K). HEK293T cells were grown in DMEM supplemented with 10% FBS. Transient transfections were performed with Transfectin reagent (Biorad, #1703352). Primary antibodies used were the rabbit anti-GAPDH (Cell Signaling, 2118S), rabbit anti-P-ERK1/2 (Cell Signaling, #9101), mouse anti-ERK1/2 (Cell Signaling #4696), rabbit anti-MEK1/2 (Cell Signaling, #9126S2), rabbit anti-P-MEK1/2 (Cell Signaling, #9154), mouse anti-FLAG® M2-tag (Sigma Aldrich F3165-1MG), rabbit anti-Vinculin (Cell Signaling, #4656).

**Expression constructs**

KinCon reporter: Following PCR amplification of the human MEK1 gene (MEK1: NM_002755.3) we fused it N-terminally with -F[1] and C-terminally with -F[2] of the Rluc -PCA (pcDNA3.1 backbone vector) as previously described (1, 2). We inserted interjacent 10-aa linkers. A site directed mutagenesis approach has been used to generate the MEK1 S218 Q56P, K57E, K57N, C121S and P124S constructs and S222 amino acid substitutions to alanine, glutamic acid and aspartic acid respectively. Flag-tagged MEK1: The Flag-tag was inserted C-terminally of MEK1 by PCR and cloned into the pcDNA3.1 vector using restriction enzymes. Flag-tagged BRAF-V600E: The Flag-tag was inserted N-terminally of BRAF-V600E by PCR and cloned into the pcDNA3.1 vector using restriction enzymes.

**Luciferase PCA analyses**

HEK293T cells were grown in DMEM supplemented with 10% FBS. Indicated versions of the Rluc PCA based reporter were transiently overexpressed in 24-well format. 48 hours post-transfection the drug exposure experiments were initiated. The growth medium was partially removed and kinase inhibitors were added to reach the final concentrations as indicated in the figure legends. DMSO was added as negative control. For the luciferase PCA measurements the growth medium was carefully removed and the cells were washed with ice cold 1xPBS. Cell suspensions were transferred to 96-well plates and subjected to luminescence analysis using the PHERAstar FSX (BMG labtech). Luciferase luminescence signals were integrated for 10 seconds following addition of the Rluc substrate h-coelenterazine (Nanolight, #301).

**Preparation of cell lysates**

Indicated constructs were overexpressed in HEK293T cells and cultured as described before. 48 hours post transfection the medium was aspirated, cells were washed with cold 1xPBS and lysed in RIPA lysis buffer. Finally, 5x SDS loading buffer was added to reach a final concentration of 1x SDS LB. To assess the protein level of over-expressed MEK1 KinCon reporters, cells were pooled following the bioluminescence measurement and lysed as described before. Prior to western blot analysis the samples were heated to 95°C for 5-10 min.

**ERK phosphorylation**

Following overexpression of indicated flag tagged MEK1 constructs in HEK293T cells we directly determined the phosphorylation status of ERK1/2 with indicated antibodies. Cell lysates were prepared as described before. A densiometric analysis using the software ImageJ was carried out to determine the protein level of pERK1/2 and ERK1/2 respectively.

**Statistical analyses**

The data were analyzed using GraphPad Prism 8.0. Log2 transformations of data was also carried out using GraphPad Prism 8.0 to ensure correct error propagation. One-sample t-tests were used to evaluate statistical significance. Values are expressed as the mean ±SEM as indicated. Significance was set at the 95% confidence level and ranked as *p<0.05, **p<0.01, ***p<0.001.

The Min test was used to evaluate the possible synergistic effect of a drug combination relative to the effects of the combination’s constituent drugs. The Min test was developed for testing synergy in fixed-dose combination clinical trials and has been adapted to multi-dose regimes (3, 4). Therefore, we used normalized fold-change RLU measurements of MEK1/BRAF-V660E treated with several drug combinations as drug response data to quantify any synergistic effect between tested groups of compounds.

**Molecular Dynamics Preparation**

Apo MEK1 models were derived from PDB structures 1SJ9 and 3EQI, while ligand-bound systems were prepared using relevant crystal structures (4U7Z, 4LMN, and 3E8N for MEK1:G805, MEK1:GDC, and MEK1:RDEA respectively). Ligand parameters were described using the CGenFF server (version 3.0.1) and apo MEK1 and ligand-bound models were submitted to the H++ server (version 3.0) to determine protonation states of histidine residues at neutral pH (5, 6). MEK1 mutants were produced with PyMOL’s mutagenesis tool (Delano, W.L., 2002). All other ionizable residues were set to their default charged state. No cysteine residues are involved in disulfide bond formation. In all, thirty-two unique systems were prepared: 4 complex states (Apo, G805, GDC0973, and RDEA119) with each complex possessing one of 7 mutant types (Q56P, K57E, C121S, P124S, S218A/S222A, S218D/S222D, and S218E/S222E) as well as the native wild type (WT). All systems are ATP bound with coordinated Mg2+ ion constructed by superimposing ANP and the Mg2+ ion from PDB 4U7Z onto a given MEK1 model and converting the N3B nitrogen atom to oxygen. Protein interactions were modeled using the CHARMM36 force field (7). All systems were solvated in a 0.15 M sodium chloride solution containing approximately 17,000 TIP3P water molecules. Solvation resulted in rectangular box sizes of approximately 8.2 × 8.2 × 8.2 nm.

**Simulation Setup**

All systems were equilibrated by performing 5000 steps of steepest descent minimization followed by 200 ps NpT ensemble simulations with gradually decreasing position restraints on the protein and ligand heavy atoms. All simulations were performed using GROMACS 2019.3. Electrostatic interactions were described using particle mesh Ewald (8). Van-der-Waals and Ewald cut-offs were set to 1.1 nm. Bonds to hydrogen atoms were constrained with the LINCS algorithm, allowing an integration time step of 2 fs. Temperature was controlled for distinct coupling groups of solvent and solute using separate v-rescale thermostats (9) at 303.15 K, using a coupling constant τ of 1 ps. An isotropic Parrinello-Rahman barostat (10) maintained a pressure of 1 atm, using a coupling constant τ of 12 ps. Following equilibration, all systems were simulated for approximately 400 nanoseconds in the NpT ensemble. Frames were saved every 50 ps, yielding a single, continuous trajectory of at least 6800 frames for each system.

**Simulation Analysis**

The trajectory of each system was fit to its reference Cα atoms with trjconv from the GROMACS 2019.3 package using the fit flag with rot+trans option. Backbone root-mean-square deviation (RMSD) was calculated with *rms* to determine the time point at which each system reaches equilibrium. All systems achieved equilibrium around 100 ns which was taken as the starting time point for subsequent data analyses. MEK1 solvent accessible surface area (SASA), RMSD, and radius of gyration (Rg) were performed on the fitted and truncated trajectory and measured using GROMACS modules *sasa, rms,* and *gyrate* respectively.

Table S1. Complete statistical parameters of the Min test for all tested single agents vs. corresponding drug combinations related to Figure 2 Panel D: Impact of MEKi and BRAFi combinations on the wt MEK1- KinCon in the presence of BRAF-V600E or mock.

|  | Rluc[F1]-MEK1-wt-Rluc[F2] + mock | | | | | |
| --- | --- | --- | --- | --- | --- | --- |
| Drug  combintaion | Vemurafenib + Cobimetinib | | Dabrafenib + Trametinib | | Encorafenib + Binimetinib | |
| Treatment  duration | 1h | 3h | 1h | 3h | 1h | 3h |
| p-value | 0,8503 | 0,9999 | 0,4628 | 0,9081 | 0,9997 | 0,9756 |
| T | -1,04 | -3,76 | 0,09 | -1,33 | -3,41 | -1,97 |
| Synergy | no | no | no | no | no | no |
|  | | | | | | |
|  |  |  |  |  |  |  |
|  | Rluc[F1]-MEK1-wt-Rluc[F2] + BRAF-V600E | | | | | |
| Drug  combintaion | Vemurafenib + Cobimetinib | | Dabrafenib + Trametinib | | Encorafenib + Binimetinib | |
| Treatment  duration | 1h | 3h | 1h | 3h | 1h | 3h |
| p-value | 0,7656 | 0,0624 | 0,021 | 0,5475 | 0,0036 | 0,0005 |
| T | -0,72 | 1,53 | 2,03 | -0,12 | 2,69 | 3,29 |
| Synergy | no | no | yes | no | yes | yes |

**SI References**

1. R. Röck *et al.*, BRAF inhibitors promote intermediate BRAF (V600E) conformations and binary interactions with activated RAS. *Science advances* **5**, eaav8463 (2019).

2. J. E. Mayrhofer *et al.*, Mutation-oriented profiling of autoinhibitory kinase conformations predicts RAF inhibitor efficacies. *Proceedings of the National Academy of Sciences* **117**, 31105-31113 (2020).

3. S. Saha, W. Brannath, B. Bornkamp, Testing multiple dose combinations in clinical trials. *Statistical Methods in Medical Research* **29**, 1799-1817 (2020).

4. J. N. Soulakova, On identifying effective and superior drug combinations via Holm's procedure based on the Min tests. *Journal of Biopharmaceutical Statistics* **19**, 280-291 (2009).

5. J. C. Gordon *et al.*, H++: a server for estimating p K as and adding missing hydrogens to macromolecules. *Nucleic Acids Res.* **33**, W368-W371 (2005).

6. J. Huang, A. D. MacKerell Jr, CHARMM36 all‐atom additive protein force field: Validation based on comparison to NMR data. *J. Comput. Chem.* **34**, 2135-2145 (2013).

7. T. Darden, D. York, L. Pedersen, Particle mesh Ewald: An N⋅ log (N) method for Ewald sums in large systems. *The Journal of chemical physics* **98**, 10089-10092 (1993).

8. G. Bussi, D. Donadio, M. Parrinello, Canonical sampling through velocity rescaling. *The Journal of chemical physics* **126**, 014101 (2007).

9. N. Kannan, A. F. Neuwald, Did protein kinase regulatory mechanisms evolve through elaboration of a simple structural component? *J. Mol. Biol.* **351**, 956-972 (2005).

10. T. D. Goddard *et al.*, UCSF ChimeraX: Meeting modern challenges in visualization and analysis. *Protein Sci.* **27**, 14-25 (2018).
